# A global view of severe maternal morbidity: moving beyond maternal mortality

**DOI:** 10.1186/s12978-018-0527-2

**Published:** 2018-06-22

**Authors:** Stacie E. Geller, Abigail R. Koch, Caitlin E. Garland, E. Jane MacDonald, Francesca Storey, Beverley Lawton

**Affiliations:** 1 Departments of Obstetrics & Gynecology and Medicine, University of Illinois at Chicago College of Medicine, Chicago, IL USA; 2 Center for Research on Women and Gender, University of Illinois at Chicago College of Medicine, Chicago, IL USA; 3 Centre for Women’s Health Research, Victoria University of Wellington, Wellington, New Zealand

## Abstract

**Background:**

Maternal mortality continues to be of great public health importance, however for each woman who dies as the direct or indirect result of pregnancy, many more women experience life-threatening complications. The global burden of severe maternal morbidity (SMM) is not known, but the World Bank estimates that it is increasing over time. Consistent with rates of maternal mortality, SMM rates are higher in low- and middle-income countries (LMICs) than in high-income countries (HICs).

**Severe maternal morbidity in high-income countries:**

Since the WHO recommended that HICs with low maternal mortality ratios begin to examine SMM to identify systems failures and intervention priorities, researchers in many HICs have turned their attention to SMM. Where surveillance has been conducted, the most common etiologies of SMM have been major obstetric hemorrhage and hypertensive disorders. Of the countries that have conducted SMM reviews, the most common preventable factors were provider-related, specifically failure to identify “high risk” status, delays in diagnosis, and delays in treatment.

**Severe maternal morbidity in low and middle income countries:**

The highest burden of SMM is in Sub-Saharan Africa, where estimates of SMM are as high as 198 per 1000 live births. Hemorrhage and hypertensive disorders are the leading conditions contributing to SMM across all regions. Case reviews are rare, but have revealed patterns of substandard maternal health care and suboptimal use of evidence-based strategies to prevent and treat morbidity.

**Effects of SMM on delivery outcomes and infants:**

Severe maternal morbidity not only puts the woman’s life at risk, her fetus/neonate may suffer consequences of morbidity and mortality as well. Adverse delivery outcomes occur at a higher frequency among women with SMM. Reducing preventable severe maternal morbidity not only reduces the potential for maternal mortality but also improves the health and well-being of the newborn.

**Conclusion:**

Increasing global maternal morbidity is a failure to achieve broad public health goals of improved women’s and infants’ health. It is incumbent upon all countries to implement surveillance initiatives to understand the burden of severe morbidity and to implement review processes for assessing potential preventability.

## Background

Maternal mortality is a sentinel event used globally to monitor maternal health, the general quality of reproductive health care, and the progress countries have made toward international development goals [1, 2]. Globally, the maternal mortality ratio (MMR) dropped from 385 maternal deaths per 100,000 live births in 1990 to 216 in 2015, a 44% reduction [3]. Most high income countries (HICs) have low maternal death rates, generally ranging from 3 to 12 per 100,000, that have consistently decreased in the last 25 years [4]. The United States is an exception with an MMR of 14 per 100,000, a 16.7% increase since 1990 [4]. Low and middle incomes countries (LMICs) still bear 99% of the burden of maternal mortality and the majority of deaths occur in sub-Saharan Africa [3] A Sustainable Development Goal for 2030 is to reduce the global MMR to 70 per 100,000 births and for no country to exceed two times that ratio (140 per 100,000).

Globally, more than half of maternal deaths between 2003 and 2009 were due to hemorrhage, hypertensive disorders, and sepsis [5]. Common causes of maternal mortality varied by region: in Northern Africa, 36.9% of deaths were due to hemorrhage compared with 16.3% in HICs [5]. Deaths due to hypertensive disorders were most common in Latin America and the Caribbean, accounting for 22.1% of deaths [5]. The vast majority of deaths due to sepsis were in LMICs [5].

Maternal mortality continues to be of great public health importance, however for each woman who dies as the direct or indirect result of pregnancy, many more women experience life-threatening complications [6, 7]. It is estimated that 50–100 women experience severe morbidity (SMM) compared to every maternal death in the United States and the rate has more than doubled from 74 per 10,000 delivery hospitalizations in 1998–99 to 163 in 2010–11 [2, 7]. Consistent with rates of maternal mortality, SMM rates are higher in LMICs than in HICs, complicating up to 8% of deliveries that take place in hospitals [8, 9]. These alarming rates and their implications for poor maternal and infant outcomes with long term poor health consequences, highlight a critical need for surveillance with the goal of understanding how to prevent SMM through quality improvement initiatives.

Maternal pregnancy outcomes can be conceptualized on a continuum of severity: normal/healthy pregnancy - > morbidity - > severe morbidity - > death [10]. Women with severe maternal morbidity experience severe pregnancy, delivery, and postpartum complications such as massive hemorrhage, cardiac arrest, organ system failure, stroke, and other health problems that may result in extended hospital stay, massive transfusion, hysterectomy, major surgery, or other major medical interventions [11]. The study of SMM provides opportunities to see a fuller picture of the quality of maternity care, potentially identifying factors associated with preventing the progression along the continuum to severe morbidity or death [2, 12]. As SMM emerges as an important area of increased interest globally, it is clear that the issues and solutions in LMICs are very different from those in HICs. This review presents current literature on SMM globally, first in HICs and then in LMICs.

## Severe maternal morbidity in high-income countries

High-income countries (HICs) are increasingly focused on SMM in addition to maternal mortality [13–15]. Given the rarity of maternal mortality in HICs, routine surveillance for SMM is now recommended to monitor maternal health and quality of care [9]. Estimates of the prevalence of SMM in the HICs depend on the way SMM is defined (Table 1). EURO-PERISTAT, a 20-year collaboration of 15 European countries focused on developing indicators of perinatal health, defined SMM as a composite of the rates of eclampsia, hysterectomy for postpartum hemorrhage, ICU admission, blood transfusion, and uterine artery embolization [13].

**Table 1:** Estimates of the Prevalence of Severe Maternal Morbidity in High-Income Countries

Author (Year)	Country	Definition of SMM	Estimated Prevalence^a^	Leading Causes
Bouvier-Colle (2012) [13]	17 EU Countries	Eclampsia	0.2–1.6	
	3 EU Countries	ICU Admission	0.5–3.1	
	10 EU Countries	Blood Transfusion	0.1–11.5	
	15 EU Countries	Hysterectomy	0.2–1.0	
	7 EU Countries	Embolisation	0.0–0.3	
Colmorn (2015) [71]	Denmark, Finland, Iceland, Norway, and Sweden	Complete uterine rupture	5.6	
Deneux-Tharaux (2017) [16]	France	Obstetric hemorrhage, hypertensive complications, Psychiatric disorder, decompensation of preexisting condition, pulmonary embolism, sepsis, stroke, amniotic fluid embolism, other	13.9	Obstetric hemorrhage (65.2%), hypertensive conditions (21.6%)
Jayaratnam (2016) [45]	Australia	WHO criteria	4.8	Hemorrhage
Jayaratnam (2011) [72]	Australia	Antepartum hemorrhage requiring emergency surgery, PPH requiring surgery, any postnatal patient requiring surgery, severe pre-eclampsia/eclampsia/HELLP, ICU admission, shock, acute ruptured ectopic, pulmonary embolism, other conditions requiring immediate medical assessment	6.0	
Kilpatrick (2016) [43]	United States	CDC method with chart review to confirm condition was truly life-threatening	7.3	Hemorrhage, hypertensive disorders
Lawton (2016) [personal communication]	New Zealand	ICU/HDU admission	6.2	Major blood loss, pre-eclampsia, sepsis
Lyndon (2012) [73]	United States	CDC method supplemented with birth certificate data	5.8	
Main (2016) [74]	United States	“Gold standard” clinical guidelines	7.3	
Marr (2014) [40]	Scotland	Major obstetric hemorrhage, eclampsia, renal or liver dysfunction, pulmonary edema, acute respiratory distress, coma, cerebrovascular event, status epilepticus, anaphylactic shock, septicemic shock, anesthetic problem, massive pulmonary embolism, ICU/coronary care unit admission	6.1	Major obstetric hemorrhage, ICU/coronary care admission
Nair (2016) [20]	England	Acute abdomen	0.01	
		Acute renal failure	0.08	
		Acute psychosis	0.05	
		Cardiac arrest/failure or infarction	0.05	
		Cerebral edema or coma	0.01	
		DIC	0.01	
		Cerebrovascular accident	0.04	
		Major complications of anesthesia	0.06	
		Obstetric embolism (inc. AFE)	0.27	
		Shock	0.20	
		Sickle cell crisis	0.05	
		Status asthmaticus	0.02	
		Status epilepticus	0.03	
		Uterine rupture	0.48	
		Eclampsia	0.71	
		Sepsis	0.44	
		Cerebral venous thrombosis	0.003	
		Assisted ventilation including tracheostomy	0.15	
		Curettage with general anesthesia	0.01	
		Dialysis	0.01	
		Evacuation of hematoma	0.50	
		Hysterectomy	0.24	
		Procedures to reduce blood flow to uterus	0.06	
		Re-closure of disrupted cesarean section wound	0.31	
		Repair of bladder or cystostomy	0.31	
		Repair of intestine	0.008	
O’Malley (2016) [75]	Ireland	WHO criteria	3.6	Hemorrhage
		Scottish Audit of SMM criteria	18.4	Hypertension
Ozimek (2016) [37]	United States	“Gold standard” clinical guidelines from Main (2016)	9.2	Hemorrhage, preeclampsia/eclampsia
Zanconato (2012) [44]	Italy	ICU admission, transfusion ≥4 units, emergency peripartum hysterectomy, arterial embolization	8.5	Hypertensive disorders, hemorrhage, sepsis
Zwart (2010) [76]	The Netherlands	ICU admission, eclampsia/HELLP syndrome, uterine rupture, major hemorrhage, miscellaneous	7.1 overall 6.3 Western ethn 8.4 non-Western ethn	
		Peripartum hysterectomy	3.5	
		Abnormally invasive placenta	4.6	
		Severe hemorrhage at delivery	11.6	

^a^Per 1000 live births

More recently, the EPIMOMS study group in France proposed a comprehensive set of 17 indicators specifically for use in HICs [16]. Their definition includes the EURO-PERISTAT indicators as well as measures of organ system dysfunction defined by minimal management-based criteria [16]. Both the EURO-PERISTAT and EPIMOMS definitions are based on data from existing sources such as hospital administrative records and laboratory tests. Similarly, in the United States, the CDC has published a list of 18 indicators and corresponding ICD codes using the 10th revision of the International Classification of Disease (ICD-10) to facilitate the identification of SMM using hospital discharge data [17, 18].

To move beyond a list of indicators and to define a composite indicator that could easily identify SMM from routinely collected population health data, researchers in Australia began with a list of 86 diagnoses and procedures that could potentially be included in the final composite [19]. To refine the components of the indicator, a validation study was conducted to assess whether cases that screened positive for SMM were true cases based on medical record review. The final SMM indicator comprised 14 diagnoses and 11 procedures with a positive predictive value of 94.6%, sensitivity of 78.4%, and specificity of 99.9% for confirmed SMM as identified by medical record review, the gold standard definition [19]. This indicator was adapted for use in England, taking into account limitations of the quality and reliability of English hospital data [20].

Departing from efforts to define SMM using information available in routinely collected administrative data, representatives from the 13 HICs in the International Network of Obstetric Surveillance Systems (INOSS) developed consensus definitions for eight severe morbidity conditions: eclampsia, amniotic fluid embolism, pregnancy-related hysterectomy, severe primary postpartum hemorrhage, uterine rupture, abnormally invasive placentation, spontaneous hemoperitoneum in pregnancy, and cardiac arrest in pregnancy [21]. Multidisciplinary panels used an iterative process to produce standardized definitions to promote comparability across countries. Clinical data is required to apply these definitions, necessitating additional data collection systems for their use [21].

Case review has long been the gold standard for assessing maternal deaths for the underlying cause of death, the factors that contributed to the progression from morbidity to death, and to determine whether the death was potentially preventable [22–25]. Reviews for potential preventability have provided valuable insights into opportunities to improve obstetrical care and management and identify themes and trends in preventability factors and translate these findings into action [26–30]. As attention in HICs moves to SMM, there have been initiatives to institute SMM case review as well [15, 24, 31]. For the purposes of case assessment, preventability can be defined as “any action or inaction on the part of the health care provider, system, patient, or a combination of these factors that may have caused progression to more severe morbidity” [32]. In other words, did the woman have to get as sick as she did? In addition to identifying factors that contributed to the progression to severe morbidity, SMM reviews may assist facilities in recognizing evidence-based practices that prevent maternal death, as women with SMM may survive because of medical intervention and best clinical practice.

The US Centers for Disease Control and Prevention (CDC) and the American College of Obstetricians and Gynecologists (ACOG) have called for greater monitoring and review of severe pregnancy and delivery complications, and also provided detailed recommendations for doing so [11, 15, 31]. The CDC and ACOG specifically recommend facility-level multidisciplinary review of all cases by using a two-factor scoring system that identifies SMM cases by: (1) admission to the intensive care unit (ICU) and/or (2) transfusion of four or more units of blood products at any time from conception through 42 days postpartum [33–35]. This 2-factor scoring system developed by Geller et al. has been validated and can be used in real time in hospital settings, unlike administrative datasets used for population-level surveillance [33, 34, 36]. To date, SMM reviews have been implemented in individual facilities in California [37] and Illinois recently piloted a statewide implementation through its regionalized perinatal system [38].

New Zealand adapted the Illinois model for their research and, with support from the New Zealand Ministry of Health, implemented multidisciplinary regional panels across the country to review cases of all women admitted to an ICU or high-dependency unit who were pregnant or within 42 days of delivery. The national rate of women with SMM admitted to an ICU/HDU was 6.2 per 1000 live births. Of those 399 cases reviewed, 34% were deemed potentially preventable, 29.5% were classified as not preventable but improvement in care was needed. Factors associated with preventable SMM cases, were provider (clinician) related in almost all cases (93.4%), most often issues related to diagnosis (inappropriate or delay in diagnosis or failure to recognize “high risk” patient) and/or treatment (inappropriate, delay or failure to treat). Major blood loss, pre-eclampsia and sepsis were the commonest clinical conditions where the severity of morbidity was deemed potentially preventable [14].

The UK implemented national reviews of SMM cases by adding it to their longstanding Confidential Enquiry into Maternal Deaths program [39]. Nominated reporting clinicians complete a monthly survey that is entered into a dedicated data collection system. The project does not provide population-level surveillance for a standardized definition of SMM; instead, it focuses on a changing set of severe morbidity/near miss conditions such as uterine rupture, eclampsia and pulmonary embolism to answer specific clinical questions [39]. Anonymous cases are reviewed by multidisciplinary experts to identify public health, hospital, and system problems that can inform future improvements in care [39].

Similarly, the Scottish Confidential Audit of Severe Maternal Morbidity (SCASMM) implemented a national 10 year surveillance project from 2003 to 2012 [40, 41]. All cases meeting one or more of the 14 SMM definitions were reported to the SCASMM during that time; cases of major obstetric hemorrhage (MOH) and eclampsia were reviewed in detail. Over the course of the project, the proportion of women with MOH who received appropriate care rose from 60% in 2004 to 80% in 2011 [41]. They found that the outcome could have been more favorable in just 4 to 10% of hemorrhage cases. Among 108 eclampsia cases that were assessed during the project period, 7 (6.5%) were deemed to have received suboptimal care [41].

The Netherlands also introduced SMM case reviews (67 cases) between 2005 and 2008 [42]. Cases were defined by ICU admission, uterine rupture, eclampsia/HELLP syndrome, massive obstetric hemorrhage, and cases referred to the panel by the treating obstetrician despite not being any of the specific criteria. Panel members were multidisciplinary and included members of the national maternal mortality review committee and clinicians of all obstetric disciplines. Substandard care and other potentially preventable factors were identified in 53 (74.6%) cases. The majority of factors identified (76.3%) were provider-related, 17.7% were health care system-related, and 6.0% were patient-related. The most common preventable factors were delays in diagnosis and treatment.

Since the WHO recommended that HICs with low maternal mortality ratios should begin to examine SMM to identify systems failures and intervention priorities [9], researchers in many HICs have turned their attention to SMM. Where surveillance has been conducted, the most common etiologies of SMM have been major obstetric hemorrhage and hypertensive disorders [13, 16, 37, 43–45]. Fewer countries have undertaken review of SMM to identify preventable factors and opportunities for improvements in maternity care provided by hospitals and health systems. Of those that have conducted SMM reviews, the most common preventable factors were provider-related, specifically failure to identify that the woman was progressing in severity, delays in diagnosis, and delays in treatment [14, 42, 46, 47].

## Severe maternal morbidity in low and middle income countries

There has also been an increased interest in SMM in low and middle income countries (LMIC) in recent years, with studies in Sub-Saharan Africa (Table 2), Middle East (Table 3), Asia (Table 4) and Latin America (Table 5) estimating their SMM burden.

**Table 2:** Estimates of the Prevalence of Severe Maternal Morbidity in Sub-Saharan Africa

Article	Country	Setting	Definition of SMM	Estimated Prevalence^a^	Leading Causes
Adeoye 2013 [66]	Nigeria	1 tertiary referral hospital, Ile-Ife	Filippi et al. 2005	109.9^b^	Hemorrhage, hypertensive disorders, dystocia
Ali 2011 [77]	Sudan	1 tertiary referral hospital, Kalassa	Filippi et al. 2005	22.1	Hemorrhage, infection, hypertensive disorders
David 2014 [78]	Mozambique	5 health facilities, Maputo city/province	eclampsia, infection hypertension, anemia, dystocia	20.2	Hemorrhage, hypertensive disorders, infection
Gebrehiwot 2014 [59]	Ethiopia	10 public hospitals	hypertensive disorders, obstetric hemorrhage, dystocia, infection, anemia	90.8	Dystocia or uterine rupture, hypertensive disorders, hemorrhage
Goldenberg 2017 [51]	Democratic Republic of Congo	14 health centers and 3 hospitals, Equateur province	Modified WHO	37.3^b^	Not reported by country
Goldenberg 2017	Kenya	23 health facilities and 3 referral hospitals, Busia, Bungoma and Kakamega counties	Modified WHO	31.2^b^	Not reported by country
Goldenberg 2017	Zambia	8 health posts, 3 district hospitals and 1 referral hospital, Kafue and Chongwe districts	Modified WHO	13.0^b^	Not reported by country
Herklots 2017 [79]	Tanzania	Tertiary referral hospital, Zanzibar	WHO	9.0	Hemorrhage, hypertensive disorders
Kalisa 2016 [80]	Rwanda	Provincial referral hospital, Musanze district	Modified WHO	21.5	Hemorrhage, hypertensive disorders
Kiruja 2017 [81]	Somaliland	Main referral hospital	WHO	88.6	Hemorrhage, hypertensive disorders, infection
Litorp 2014 [82]	Tanzania	2 hospitals, Dar es Salaam	WHO	36	Hypertensive disorders, hemorrhage
Liyew 2017 [83]	Ethiopia	5 public hospitals, Addis Ababa	WHO	8.1	Hypertensive disorders, hemorrhage, abortive outcome
Lori 2012 [62]	Liberia	Rural county	Modified WHO and Filippi et al. 2005	16% of deliveries	Hemorrhage, anemia, sepsis
Mbachu 2017 [54]	Nigeria	Private hospital, Elele	WHO	198	Hemorrhage, abortive outcome, hypertensive disorders
Mekango 2017 [84]	Ethiopia	6 public hospitals, Tigray	Filippi et al. 2005	101	Hemorrhage, hypertensive disorders, dystocia
Nakimuli 2016 [85]	Uganda	2 referral hospitals, Central Uganda	WHO	8.42	Hypertensive disorders, hemorrhage
Nelissen 2013 [86]	Tanzania	Referral hospital, rural	Modified WHO	23.6	Hemorrhage, abortive outcome, dystocia
Oladapo 2016 [57]	Nigeria	42 public tertiary hospitals	WHO	15.8	Hemorrhage, hypertensive disorders, abortive outcome
Rulisa 2015 [87]	Rwanda	University hospital, Kigali	WHO	8	Sepsis, hypertensive disorders, hemorrhage
Sayinzoga 2017 [88]	Rwanda	4 rural district hospitals	Modified WHO	36	Hemorrhage, uterine rupture, abortive outcome
Soma-Pillay 2015 [89]	South Africa	9 delivery facilities, Gauteng province	WHO	4.4^c^	Hemorrhage, hypertensive disorders, sepsis
Tuncalp 2013 [90]	Ghana	Tertiary referral hospital, Accra	WHO	28.6	Not reported

^a^per 1000 live births

^b^per 1000 deliveries

^c^per 1000 pregnancies

**Table 3:** Estimates of the Prevalence of Severe Maternal Morbidity in North Africa and Middle East

Article	Country	Setting	Definition of SMM	Estimated Prevalence^a^	Leading Causes
Akrawi 2017 [91]	Iraq	Public tertiary hospital, Erbil City	Modified WHO	8.2	Hypertensive disorders, hemorrhage
Assarag 2015 [92]	Morocco	3 public referral hospital, Marrakech	Sahel et al. 2011	12	Hemorrhage
Bashour 2015 [93]	Egypt	Public tertiary hospital, Cairo	WHO	12.1	Hemorrhage
Bashour 2015	Lebanon	Public hospital, Beirut	WHO	4.3	Hemorrhage
Bashour 2015	Palestine	Public referral hospital, Ramallah	WHO	12.9	Hemorrhage
Bashour 2015	Syria	University hospital, Damascus	WHO	4.5	Hemorrhage
Ghardallou 2016 [94]	Tunisia	Public tertiary hospital, Sousse	WHO	5.86	Hemorrhage, hypertensive disorders
Ghazivakili 2016 [95]	Iran	13 public and private hospital, Alborz province	WHO	4.97	Hypertensive disorders, hemorrhage
Jabir 2013 [63]	Iraq	6 public hospital, Baghdad	WHO	5.06	Hemorrhage, hypertensive disorders

^a^per 1000 live births

**Table 4:** Estimates of the Prevalence of Severe Maternal Morbidity in Asia

Article	Country	Setting	Definition of SMM	Estimated Prevalence^a^	Leading Causes
Bolnga 2017 [96]	Papua New Guinea	Provincial hospital, Madang Province	Modified WHO	25.4	Hemorrhage
Goldenberg 2017 [51]	India	18 primary health centers, 3 tertiary hospitals and 8 secondary hospitals Belagavi	Modified WHO	28.1^b^	Not reported by country
Goldenberg 2017	India	20 primary health centers, 10 tertiary hospitals and 129 secondary hospitals, Nagpur	Modified WHO	4.4^b^	Not reported by country
Goldenberg 2017	Pakistan	47 primary health clinics, 25 secondary care facilities and 3 referral hospitals, Thatta district	Modified WHO	81.9^b^	Not reported by country
Kalra 2014 [97]	India	Tertiary hospital, Rajasthan	Geller et al. 2004	4.8	Hemorrhage, hypertensive disorders
Khan 2017 [98]	India	Tertiary referral hospital, New Delhi	Geller et al. 2004, Pattinson et al. 2003, ICD-10	14	Hemorrhage, hypertensive disorders, anemia
Luexay 2014 [99]	Laos	Community survey, Sayaboury province	WHO	9.8	Hemorrhage, hypertensive disorders
Mazhar 2015 [100]	Pakistan	16 government hospitals	WHO	7.0	Hemorrhage, hypertensive disorders, uterine rupture
Norhayati 2016 [101]	Malaysia	2 tertiary hospitals, Kelantan	WHO	2.2	Hemorrhage, hypertensive disorders
Roopa 2013 [102]	India	Tertiary referral hospital, Manipal	WHO	17.8	Hemorrhage, hypertensive disorders, sepsis
Pandey 2014 [55]	India	Medical college hospital, Uttar Predesh	WHO	120	Hemorrhage, hypertensive disorders, anemia
Purandare 2014 [60]	India	6 medical college hospitals	Pregnancy-specific disorders. Pre-existing disorders aggravated during pregnancy, Pregnancy-specific medical disorders, Incidental and accidental causes that occurred in pregnancy	9.6	Hemorrhage
Rana 2013 [103]	Nepal	9 tertiary hospitals, Kathmandu	WHO	3.8	Hemorrhage, hypertensive disorders
Shen 2013 [104]	China	Private tertiary hospital, Suzhou	WHO	4	Hemorrhage, hypertensive disorders
Shrestha 2010 [105]	Nepal	Tertiary hospital, Kathmandu	Geller et al. 2004	23.1^b^	Hemorrhage, hypertensive disorders
Siddiqui 2012 [106]	Pakistan	Public tertiary hospital, Karachi	Modified Waterstone et al. 2001	77	Hemorrhage, hypertensive disorders, uterine rupture
Tan 2015 [107]	China	8 hospital, Sichuan province	Hemorrhage, hypertensive disorders, uterine rupture, interventional radiology, blood transfusions, laparotomy, ICU admission, multiple organ dysfunction syndromes	43.4	Did not report
Tanimia 2016 [108]	Papua New Guinea	National referral hospital, Port Moresby	Modified WHO	9.1	Hemorrhage, hypertensive disorders, infection

^a^per 1000 live births

^b^per 1000 deliveries

**Table 5:** Estimates of the Prevalence of Severe Maternal Morbidity in Latin America

Article	Country	Setting	Definition of SMM	Estimated Prevalence^a^	Leading Causes
De Mucio 2016 [109]	Argentina	3 hospitals	WHO	2.62	Not reported
De Mucio 2016	Colombia	1 hospital	WHO	8.98	Not reported
De Mucio 2016	Dominican Republic	1 hospital	WHO	22.56	Not reported
De Mucio 2016	Ecuador	1 hospital	WHO	8.77	Not reported
De Mucio 2016	Honduras	2 hospitals	WHO	16.31	Not reported
De Mucio 2016	Nicaragua	1 hospital	WHO	8.39	Not reported
De Mucio 2016	Paraguay	1 hospital	WHO	5.99	Not reported
De Mucio 2016	Peru	1 hospital	WHO	34.92	Not reported
Dias 2014 [110]	Brazil	Birth in Brazil national survey	WHO	10.21	Not reported
Goldenberg 2017 [51]	Guatemala	1 referral hospital, 30 health centers, and 42 health posts, Chimaltenango region	Modified WHO	61.1^b^	Not reported by country
Karolinski 2013 [58]	Argentina	25 public hospitals	ICU admit, hysterectomy, organ dysfunction	8.49	Not reported
Lima 2016 [111]	Brazil	Tertiary hospital	WHO	10.8	Not reported
Madeiro 2015 [112]	Brazil	Tertiary hospital, Piaui	WHO	9.6	Hypertensive disorders, hemorrhage, infection
Galveo 2014 [113]	Brazil	2 referral hospitals, Sergipe	WHO	5.8	Hypertensive disorders, hemorrhage

^a^per 1000 live births

^b^per 1000 deliveries

There is no standardized SMM definition; at least seven different definitions are used in LMICs as well as several individual studies that used their own definition. Of these, the World Health Organization’s (WHO) definition is the most commonly used. The WHO defines SMM as “a woman who nearly died but survived a complication that occurred during pregnancy, birth or within 42 days of termination of pregnancy” [48]. The WHO prefers to use the term “maternal near miss” to describe these women. A maternal near miss is identified when a woman develops one or more signs of organ dysfunction as described by 25 clinical, laboratory, or management criteria [48]. However, the applicability of the WHO criteria to low resource settings where certain laboratory and management tests/procedures are not routinely available is disputed and many countries must modify the WHO criteria for their settings [49–51]. For example, a study in Ethiopia modified the definition of SMM to include any woman who received 1 or more units of blood instead of 5 or more units of blood as the WHO suggests [51, 52]. The Global Network also modified the WHO definition by adding transfusion of any volume and excluding all WHO laboratory criteria for their definition of SMM [51]. The other definitions utilized in LMICs, such as Geller et al. [33] and Filippi et al. [53] are much simpler than the WHO criteria and generally use clinical or management criteria, such as diagnosis of eclampsia or emergency hysterectomy to identify SMM.

It is difficult to compare SMM across countries due to the heterogeneity of SMM definitions. However, it is clear that the highest burden of SMM is in Sub-Saharan Africa, where estimates of SMM are as high as 198 per 1000 live births [54]. Asia also has a high SMM burden, with one study in India reporting a SMM rate of 120 per 1000 live births [55]. Hemorrhage and hypertensive disorders are the leading conditions contributing to SMM across all regions. These trends mirror maternal mortality trends, underscoring the importance of studying SMM.

Given the burden of collecting data on maternal deaths and reviewing these cases for potential preventability, there are large regions of the world such as Central Asia or Central Africa where there are no SMM estimates and we were not able to find any LMICs conducting national surveillance of SMM. However, Brazil is progressing towards a national surveillance system with the Brazilian Network for Surveillance of Severe Maternal Morbidity, which identifies SMM cases in 27 hospitals throughout the country [56]. Additionally, the Global Network Near-Miss Mortality System is conducting population-based surveillance of SMM at seven district/province level sites in Africa, Asia and Central America [51]. The majority of SMM studies that do take place occur in a single hospital, a single city or a single province/state and only rarely include multiple regions [57–60] This leads to vast differences in reported SMM rates between studies in the same country, such as the rate of SMM ranging from 9.6 to 120 per 1000 live births in two studies from India [55, 60].

The few studies of SMM case reviews that have been reported in LMICs include Ethiopia, Liberia, India and Moldova [59–62]. Overall, these studies incorporated a range of disease-specific, and pregnancy-specific clinical criteria to identify cases of SMM. India and Ethiopia found similar factors that contributed to SMM, such as lack of prenatal care, inability to access services, delays in seeking care, lack of medical equipment/supplies and health personnel issues [59, 60]. Liberia’s analysis focused only on understanding delays in seeking and receiving care [62]. Ethiopia, India and Moldova found that the review process is feasible and that providers were more accepting of SMM reviews compared to mortality reviews because they felt the process did not assign blame and they could highlight instances where they had provided excellent care and saved the woman’s life [59–61].

Globally, the pattern is emerging that substandard maternal health care and suboptimal use of evidence-based strategies to prevent and treat morbidity are common across many countries regardless of wealth, contributing to the high burden of SMM [52, 58, 63, 64]. Preventability reviews of SMM have the potential to dramatically improve maternal health but few LMICs have conducted SMM reviews and they did not utilize a preventability framework. The lack of surveillance and review in countries with the highest burden of SMM and maternal death only perpetuates the poor maternal health outcomes observed in these regions. Currently, the state of SMM surveillance reflects the broad disinvestment in maternal health, as a standardized definition that is globally applicable is elusive and large regions of world have no SMM estimates.

## Effects of SMM on delivery outcomes and infants

Severe maternal morbidity not only puts the woman’s life at risk, her fetus/neonate may suffer consequences of morbidity and mortality as well. Preventing a woman’s progression along the continuum of severity may also improve delivery outcomes and newborn health. If we incorporate delivery outcomes, the expanded continuum includes both mother and child: normal/healthy pregnancy - > morbidity - > severe morbidity - > death - > delivery outcome - > neonatal morbidity.

Adverse delivery outcomes such as fetal death, NICU admission, preterm birth, 5-min Apgar score less than 7 and low birth weight occur at a higher frequency among women with SMM (Table 6). A nationwide study in New Zealand found that 49.4% of women with SMM suffered one or more of these adverse delivery outcomes. Preterm birth is significantly associated with SMM, with between 22 and 41% of women with SMM having a preterm birth [65, 66]. Interestingly, HICs and LMICs report similar rates of preterm birth among women with SMM. Neonatal intensive care unit (NICU) admission rates are also high among women with SMM. These rates are higher in high and middle-income countries, which likely reflect the lack of availability of NICUs in low-income countries. SMM significantly increases the odds of a fetal death in both HICs and LMICs [65, 67]. Many of these adverse delivery outcomes are associated with the woman having preeclampsia and a need for delivery as her disease process progresses [43, 68].

**Table 6:** Adverse Delivery Outcomes among Women with SMM

Author	City/State, Country	SMM Definition	Adverse Delivery outcome^a^	Estimated Prevalence^b^
Adeoye 2013 [66]	Ile-Ife, Nigeria	Filippi et al. 2005	Fetal death	28.4
			Low birth weight	44.4
			Preterm^c^	41.3
Koch [38]	Illinois, United States	ICU admission, ≥4 units packed red blood cells	Fetal death	8.9
			NICU	39.7
			Apgar < 7	16.9
			Low birth weight	31.2
			Preterm	38.1
Lawton 2017 [personal communication]	New Zealand	ICU/HDU admission	Fetal death	5.1
			NICU	44.1
			Preterm	38.5
Jakobsson 2015 [65]	Finland	abnormally invasive placenta, uterine rupture, emergency peripartum hysterectomy	Fetal death	7.5
			NICU	31.2
			Apgar < 7	19
			Low birth weight	16.1
			Preterm	22.3
Nakimuli 2015 [85]	Kampala, Uganda	WHO	Fetal death	12.0
			NICU	18.4
			Low birth weight	15.8
Nardello 2017 [68]	Aracaju, Brazil	WHO	Fetal death	8.9
			NICU	41.8
			Apgar < 7	12.5
			Low birth weight	36.7
			Preterm	38
Oliveira 2013 [114]	Recife, Brazil	WHO	Fetal death	19.5
			Apgar < 7	9.0

^a^Adverse delivery outcomes are defined as:

• 5 min Apgar score < 7

• birthweight less than 2500 g

• < 37 weeks gestational age

^b^percent of SMM cases with adverse delivery outcome

^c^ < 38 weeks gestational age at delivery

Adverse delivery outcomes are often preventable. New Zealand found that 38.8% of adverse delivery outcomes for women with SMM were preventable and suggested that better care of the woman while pregnant or during delivery could have improved the outcome. Provider (delay in timely diagnosis and treatment) and system (poor communication, failure to follow evidence-based guidelines) factors were the major preventable issues. In Scotland, audits of SMM cases were credited with the steep decline of perinatal mortality observed in Scotland between 2005 and 2012 [40]. In the UK, reviews of stillbirth and neonatal death found that nearly 80% of those deaths could have been prevented by improvements in care [69]. These findings raise the important point that reducing preventable severe maternal morbidity not only reduces the potential for maternal mortality but also improves the health and well-being of the newborn.

## A call to action

Despite gains throughout the 20^th^ century, maternal health remains a major public health concern. It is therefore critical to implement the global study of SMM through enhanced surveillance and case review to lay the foundational work to develop initiatives for quality care improvement efforts and the ability to translate these findings into policy and practice to improve the health of women and their infants. The observed increase in maternal morbidity and mortality is not only a failure to achieve broad public health goals of improved women’s health, but also contribute to sub-optimal delivery outcomes and poor infant health.

HICS generally have the resources to implement surveillance and reviews of SMM cases. This can be implemented as hospital level quality improvement initiatives or at a regional or statewide level. There are several well validated tools [33, 36] that can be utilized to identify women with severe morbidity as well as tools to conduct multidisciplinary reviews. LMICs may want to begin with surveillance efforts using a limited number of variables to estimate the significance of the issue and incorporate SMM reviews into ongoing maternal mortality reviews.

## Conclusion

Despite gains throughout the 20^th^ century, maternal health remains a major global public health concern. Of particular concern is that SMM rates appear to be trending upward [11, 70]. Such increases in maternal morbidity not only are failures to achieve broad public health goals of improved women’s health, but also contribute to sub-optimal delivery outcomes and poor infant health. Therefore, it is incumbent upon all countries to implement surveillance initiatives to understand the burden of severe morbidity and to implement review processes for assessing potential preventability. Preventing a woman’s progression along the continuum of severity may also improve delivery outcomes and newborn health. This will enable us to gather the data necessary to implement evidence-based interventions that will lead to lower rates of SMM and, ultimately, maternal mortality (MDG 5) and subsequently lower rates of preterm births and neonatal deaths (MDG 4).

## References

[1] Hogan MC, Foreman KJ, Naghavi M (2010). Maternal mortality for 181 countries, 1980-2008: a systematic analysis of progress towards millennium development goal 5. Lancet.

[2] Creanga AA, Berg CJ, Ko JY (2014). Maternal mortality and morbidity in the United States: where are we now?. J Women’s Heal..

[3] WHO, UNICEF, UNFPA (2015). Trends in maternal mortality: 1990 to 2015.

[4] WHO, UNICEF, UNFPA, et al. *Trends in maternal mortality : 1990 to 2010*. Geneva, Switzerland, 2012.

[5] Say L, Chou D, Gemmill A (2014). Global causes of maternal death: a WHO systematic analysis. Lancet Glob Heal.

[6] Creanga AA, Berg CJ, Syverson C (2015). Pregnancy-related mortality in the United States, 2006–2010. Obstet Gynecol..

[7] Grobman WA, Bailit JL, Rice MM (2014). Frequency of and factors associated with severe maternal morbidity. Obstet Gynecol..

[8] Tunçalp O, Hindin MJ, Souza JP (2012). The prevalence of maternal near miss: a systematic review. BJOG.

[9] Say L, Pattinson RC, Gülmezoglu a M (2004). WHO systematic review of maternal morbidity and mortality: the prevalence of severe acute maternal morbidity (near miss). Reprod Health.

[10] Geller SE, Rosenberg D, Cox SM (2002). Defining a conceptual framework for near-miss maternal morbidity. J Am Med Womens Assoc.

[11] Callaghan WM, Creanga AA, Kuklina EV (2012). Severe maternal morbidity among delivery and postpartum hospitalizations in the United States. Obstet Gynecol..

[12] Geller SE, Rosenberg D, Cox SM (2004). The continuum of maternal morbidity and mortality: factors associated with severity. Am J Obstet Gynecol.

[13] Bouvier-Colle M-H, Mohangoo A, Gissler M (2012). What about the mothers? An analysis of maternal mortality and morbidity in perinatal health surveillance systems in Europe. BJOG An Int J Obstet Gynaecol.

[14] Lawton B, MacDonald EJ, Brown SA (2014). Preventability of severe acute maternal morbidity. Am J Obs Gynecol.

[15] Callaghan WM, Grobman WA, Kilpatrick SJ (2014). Facility-based identification of women with severe maternal morbidity: it is time to start. Obstet Gynecol..

[16] Deneux-Tharaux C, Bouvier-Colle M-H, EPIMOMS Study Group (2017). Severe acute maternal morbidity in France: the epimoms population-based study. Am J Obstet Gynecol.

[17] Creanga A a, Berg CJ, Syverson C (2012). Race, ethnicity, and nativity differentials in pregnancy-related mortality in the United States: 1993-2006. Obstet Gynecol.

[18] Centers for Disease Control and Prevention. Severe Maternal Morbidity Indicators and Corresponding ICD Codes During Delivery Hospitalization. 2017. https://www.cdc.gov/reproductivehealth/maternalinfanthealth/smm/severe-morbidity-ICD.html. Accessed 10 Jan 2018.

[19] Roberts CL, Cameron CA, Bell JC (2008). Measuring maternal morbidity in routinely collected health data: development and validation of a maternal morbidity outcome Indicator Measu health data development and validation of a maternal morbidity outcome Indicator. Med Care.

[20] Nair M, Kurinczuk JJ, Knight M (2016). Establishing a national maternal morbidity outcome indicator in England: a population- based study using routine hospital data. PLoS One.

[21] Schaap T, Bloemenkamp K, Deneux-Tharaux C, et al. Defining definitions: a Delphi study to develop a core outcome set for conditions of severe maternal morbidity. BJOG An Int J Obstet Gynaecol. 2017:1–8. 10.1111/1471-0528.14833 28755459

[22] Kilpatrick SJ, Prentice P, Jones RL (2012). Reducing maternal deaths through state maternal mortality review. J Women’s Heal.

[23] Goodman D, Stampfel C, Creanga AA (2013). Revival of a core public health function: state- and urban-based maternal death review processes. J Women’s Heal..

[24] World Health Organization. Beyond the numbers: reviewing maternal deaths and complications to make pregnancy safer. Geneva, 2004. 10.1093/bmb/ldg009 14711752

[25] Callaghan WM (2014). State-based maternal death reviews: assessing opportunities to alter outcomes. Am J Obstet Gynecol.

[26] Wong CA, Scott S, Jones RL, et al. The state of Illinois obstetric hemorrhage project: pre-project and post-training examination scores. J Matern Neonatal Med. 2015;0:1–5. 10.3109/14767058.2015.1021672 25758626

[27] Lal AK, Geller S, Scott S (2015). Assessment of hospital readiness for obstetric hemorrhage. Evid Based Womenʼs Heal J.

[28] Shellhaas C, Conrey E (2018). State-based review of maternal Deaths: the Ohio experience. Clin Obstet Gynecol..

[29] Bacak SJ, Berg CJ (2006). State maternal mortality review: accomplishments of nine States.

[30] Mitchell C, Lawton E, Morton C (2014). California pregnancy-associated mortality review: mixed methods approach for improved case identification, cause of death analyses and translation of findings. Matern Child Health J.

[31] Kilpatrick SJ, Berg C, Bernstein P (2014). Standardized severe maternal morbidity review: rationale and process. Obstet Gynecol.

[32] Geller SE, Cox SM, Kilpatrick SJ (2006). A descriptive model of preventability in maternal morbidity and mortality. J Perinatol.

[33] Geller SE, Rosenberg D, Cox S (2004). A scoring system identified near-miss maternal morbidity during pregnancy. J Clin Epidemiol.

[34] Callaghan WM, Grobman WA, Kilpatrick SJ (2014). Facility-based identification of women with severe maternal morbidity. Obstet Gynecol..

[35] Kilpatrick SJ, Berg C, Bernstein P (2014). Standardized severe maternal morbidity review. Obstet Gynecol.

[36] You WB, Chandrasekaran S, Sullivan J (2013). Validation of a scoring system to identify women with near-miss maternal morbidity. Am J Perinatol.

[37] Ozimek JA, Eddins RM, Greene N (2016). Opportunities for improvement in care among women with severe maternal morbidity. Am J Obstet Gynecol.

[38] Koch AR, Roesch PT, Garland CE, et al. Implementing statewide severe maternal morbidity review: the Illinois experience. J Public Heal Manag Pract. 2018; 10.1097/PHH.0000000000000752. 10.1097/PHH.0000000000000752 29521849

[39] Knight M, Lewis G, Acosta CD (2014). Maternal near-miss case reviews: the UK approach. BJOG.

[40] Marr L, Lennox C, McFadyen AK (2014). Quantifying severe maternal morbidity in Scotland: a continuous audit since 2003. Curr Opin Anaesthesiol.

[41] Lennox C, Marr L, Healthcare Improvement Scotland. Scottish confidential audit of severe maternal morbidity: reducing avoidable harm. 10th Annual Report. 2014. http://www.healthcareimprovementscotland.org/our_work/reproductive,_maternal_child/programme_resources/scasmm.aspx. Accessed Feb 2016.

[42] Van Dillen J, Mesman JAJM, Zwart JJ (2010). Introducing maternal morbidity audit in the Netherlands. BJOG An Int J Obstet Gynaecol.

[43] Kilpatrick SJ, Abreo A, Gould J, et al. Confirmed severe maternal morbidity is associated with high rate of preterm delivery. Am J Obstet Gynecol. 2016:1–7. 10.1016/j.ajog.2016.02.026 26899903

[44] Zanconato G, Cavaliere E, Iacovella C (2012). Severe maternal morbidity in a tertiary care centre of northern Italy: a 5-year review. J Matern Neonatal Med..

[45] Jayaratnam S, Burton A, Connan KF (2016). Maternal ‘near miss’ at Royal Darwin Hospital: an analysis of severe maternal morbidity at an Australian regional tertiary maternity unit. Aust New Zeal J Obstet Gynaecol.

[46] Della Torre M, Kilpatrick SS, Hibbard JJ (2011). Assessing preventability for obstetric hemorrhage. Am J Perinatol.

[47] Kominiarek MA, Scott S, Koch AR (2017). Preventing maternal morbidity from obstetric hemorrhage: implications of a provider training initiative. Am J Perinatol.

[48] Say L, Souza JP, Pattinson RC (2009). Maternal near miss - towards a standard tool for monitoring quality of maternal health care. Best Pract Res Clin Obstet Gynaecol.

[49] Witteveen T, De Koning I, Bezstarosti H (2016). Validating the WHO maternal near miss tool in a high-income country. Acta Obstet Gynecol Scand.

[50] Witteveen T, Bezstarosti H, de Koning I (2017). Validating the WHO maternal near miss tool: comparing high- and low-resource settings. BMC Pregnancy Childbirth.

[51] Goldenberg RL, Saleem S, Ali S (2017). Maternal near miss in low-resource areas. Int J Gynecol Obstet.

[52] Nelissen E, Mduma E, Broerse J (2013). Applicability of the WHO maternal near miss criteria in a low-resource setting. PLoS One.

[53] Filippi V, Ronsmans C, Gohou V (2005). Maternity wards or emergency obstetric rooms? Incidence of near-miss events in African hospitals. Acta Obstet Gynecol Scand.

[54] Mbachu II, Ezeama C, Osuagwu K (2017). A cross sectional study of maternal near miss and mortality at a rural tertiary Centre in southern Nigeria. BMC Pregnancy Childbirth.

[55] Pandey A, Das V, Agarwal A (2014). Evaluation of obstetric near miss and maternal deaths in a tertiary Care Hospital in North India: shifting focus from mortality to morbidity. J Obstet Gynecol India.

[56] Cecatti JG, Costa ML, Haddad SM (2016). Network for surveillance of severe maternal morbidity: a powerful national collaboration generating data on maternal health outcomes and care. BJOG An Int J Obstet Gynaecol.

[57] Oladapo OT, Adetoro OO, Ekele BA (2016). When getting there is not enough: a nationwide cross-sectional study of 998 maternal deaths and 1451 near-misses in public tertiary hospitals in a low-income country. BJOG An Int J Obstet Gynaecol.

[58] Karolinski A, Mercer R, Micone P (2013). The epidemiology of life-threatening complications associated with reproductive process in public hospitals in Argentina. BJOG An Int J Obstet Gynaecol.

[59] Gebrehiwot Y, Tewolde BT (2014). Improving maternity care in Ethiopia through facility based review of maternal deaths and near misses. Int J Gynecol Obstet.

[60] Purandare C, Bhardwaj A, Malhotra M (2014). Maternal near-miss reviews: lessons from a pilot programme in India. BJOG.

[61] Baltag V, Filippi V, Bacci A (2012). Putting theory into practice: the introduction of obstetric near-miss case reviews in the Republic of Moldova. Int J Qual Heal Care.

[62] Lori JR, Starke AE (2012). A critical analysis of maternal morbidity and mortality in Liberia, West Africa. Midwifery.

[63] Jabir M, Abdul-Salam I, Suheil DM (2013). Maternal near miss and quality of maternal health care in Baghdad Iraq. BMC Pregnancy Childbirth.

[64] Mawarti Y, Utarini A, Hakimi M (2017). Maternal care quality in near miss and maternal mortality in an academic public tertiary hospital in Yogyakarta, Indonesia: a retrospective cohort study. BMC Pregnancy Childbirth.

[65] Jakobsson M, Tapper AM, Palomäki O (2015). Neonatal outcomes after the obstetric near-miss events uterine rupture, abnormally invasive placenta and emergency peripartum hysterectomy - prospective data from the 2009-2011 Finnish NOSS study. Acta Obstet Gynecol Scand.

[66] Adeoye IA, Onayade AA, Fatusi AO (2013). Incidence, determinants and perinatal outcomes of near miss maternal morbidity in Ile-Ife Nigeria: a prospective case control study. BMC Pregnancy and Childbirth.

[67] Firdawek E, Worku A (2015). Maternal near miss and still birth in developing countries: a systematic review with meta-analysis. J Pregnancy Child Heal.

[68] Nardello DM, Guimaraes AMDN, ID de C B (2017). Fetal and neonatal deaths of children of patients classifi ed as near miss. Rev Bras Enferm.

[69] Draper E, Kurinczuk J, Kenyon S (2017). MBRRACE-UK 2017 perinatal confidential enquiry: term, singleton, intrapartum stillbirth and intrapartum-related neonatal death.

[70] Filippi V, Chou D, Ronsmans C, et al. Levels and causes of maternal mortality and morbidity. In: Black RE, Laxminarayan R, Temmerman M, et al. (eds) Reproductive, Maternal, Newborn, and Child Health. Disease Control Priorities, 3 edition, volume 2. Washington, DC, 2016, pp. 51–70.

[71] Colmorn LB, Petersen KB, Jakobsson M (2015). The Nordic obstetric surveillance study: a study of complete uterine rupture, abnormally invasive placenta, peripartum hysterectomy, and severe blood loss at delivery. Acta Obstet Gynecol Scand.

[72] Jayaratnam S, De Costa C, Howat P. Developing an assessment tool for maternal morbidity 'near miss'--a prospective study in a large Australian regional hospital. Aust New Zeal J Obstet Gynaecol. 2011;51:421–5. 10.1111/j.1479-828X.2011.01330.x 21806590

[73] Lyndon A, Lee HC, Gilbert WM (2012). Maternal morbidity during childbirth hospitalization in California. J Matern Neonatal Med.

[74] Main EK, Abreo A, McNulty J (2016). Measuring severe maternal morbidity: validation of potential measures. Am J Obstet Gynecol.

[75] O’Malley EG, Popivanov P, Fergus A (2016). Maternal near miss: what lies beneath?. Eur J Obstet Gynecol Reprod Biol.

[76] Zwart JJ, Jonkers MD, Richters A (2011). Ethnic disparity in severe acute maternal morbidity: a nationwide cohort study in the Netherlands. Eur J Pub Health.

[77] Ali AAA, Khojali A, Okud A (2011). Maternal near-miss in a rural hospital in Sudan. BMC Pregnancy Childbirth.

[78] David E, Machungo F, Zanconato G (2014). Maternal near miss and maternal deaths in Mozambique: a cross-sectional, region-wide study of 635 consecutive cases assisted in health facilities of Maputo province. BMC Pregnancy Childbirth.

[79] Herklots T, Van Acht L, Meguid T (2017). Severe maternal morbidity in Zanzibar’s referral hospital: measuring the impact of in- hospital care. PLoS One.

[80] Kalisa R, Rulisa S, van den Akker T (2016). Maternal near miss and quality of care in a rural Rwandan hospital. BMC Pregnancy Childbirth.

[81] Kiruja J, Osman F, Egal JA (2017). Maternal near-miss and death incidences – frequencies, causes and the referral chain in Somaliland: a pilot study using the WHO near-miss approach. Sex Reprod Healthc.

[82] Litorp H, Kidanto HL, Rööst M (2014). Maternal near-miss and death and their association with caesarean section complications: a cross-sectional study at a university hospital and a regional hospital in Tanzania. BMC Pregnancy Childbirth..

[83] Liyew EF, Yalew AW, Afework MF (2017). Incidence and causes of maternal near-miss in selected hospitals of Addis Ababa thiopia. PLoS One.

[84] Mekango DE, Alemayehu M, Gebregergs GB (2017). Determinants of maternal near miss among women in public hospital maternity wards in northern Ethiopia: a facility based case-control study. PLoS One.

[85] Nakimuli A, Nakubulwa S, Kakaire O (2016). Maternal near misses from two referral hospitals in Uganda: a prospective cohort study on incidence, determinants and prognostic factors. BMC Pregnancy Childbirth.

[86] Nelissen EJT, Mduma E, Ersdal HL (2013). Maternal near miss and mortality in a rural referral hospital in northern Tanzania : a cross-sectional study. BMC Pregnancy Childbirth.

[87] Rulisa S, Umuziranenge I, Small M (2015). Maternal near miss and mortality in a tertiary care hospital in Rwanda. BMC Pregnancy Childbirth.

[88] Sayinzoga F, Bijlmakers L, van der Velden K (2017). Severe maternal outcomes and quality of care at district hospitals in Rwanda- a multicentre prospective case-control study. BMC Pregnancy Childbirth.

[89] Soma-Pillay P, Pattinson RC, Langa-Mlambo L (2015). Maternal near miss and maternal death in the Pretoria academic complex, South Africa: a population-based study. South African Med J.

[90] Tunçalp Ö, Hindin MJ, Adu-Bonsaffoh K (2013). Assessment of maternal near-miss and quality of care in a hospital-based study in Accra, Ghana. Int J Gynecol Obstet.

[91] Akrawi VS, Al-Hadithi TS, Al-Tawil NG (2017). Major determinants of maternal near-miss and mortality at the maternity teaching hospital, Erbil city, Iraq. Oman Med J.

[92] Assarag B, Dujardin B, Delamou A (2015). Determinants of maternal near-miss in Morocco: too late, too far, too sloppy?. PLoS One.

[93] Bashour H, Saad-Haddad G, DeJong J (2015). A cross sectional study of maternal ‘near-miss’ cases in major public hospitals in Egypt, Lebanon Palestine and Syria. BMC Pregnancy Childbirth.

[94] Ghardallou M, Ajmi T, Mkhazni A (2016). Maternal near miss and quality of obstetric Care in a Tunisian Teriary Level Maternity. Afr J Reprod Health.

[95] Ghazivakili Z, Lotfi R, Kabir K (2016). Maternal near miss approach to evaluate quality of care in Alborz province, Iran. Midwifery.

[96] Bolnga JW, Morris M, Totona C, et al. Maternal near-misses at a provincial hospital in Papua New Guinea: a prospective observational study. Aust New Zeal J Obstet Gynaecol. 2017:624–9. 10.1111/ajo.12650 28580650

[97] Kalra P, Kachhwaha C (2014). Obstetric near miss morbidity and maternal mortality in a tertiary care Centre in western Rajasthan. Indian J Public Heal.

[98] Khan T, Laul P, Laul A (2017). Prognostic factors of maternal near miss events and maternal deaths in a tertiary healthcare facility in India. Int J Gynecol Obstet.

[99] Luexay P, Malinee L, Pisake L (2014). Maternal near-miss and mortality in Sayaboury Province, Lao PDR. BMC Public Health.

[100] Mazhar SB, Batool A, Emanuel A (2015). Severe maternal outcomes and their predictors among Pakistani women in the WHO multicountry survey on maternal and newborn health. Int J Gynecol Obstet.

[101] Norhayati MN, Nik Hazlina NH, Sulaiman Z (2016). Severe maternal morbidity and near misses in tertiary hospitals, Kelantan, Malaysia: a cross-sectional study. BMC Public Health.

[102] Roopa P, Verma S, Rai L (2013). ‘Near miss’ obstetric events and maternal deaths in a tertiary care hospital: an audit. J Pregnancy.

[103] Rana A, Baral GDG (2013). Maternal near miss: a multicenter surveillance in Kathmandu Valley. J Nepal Med Assoc.

[104] Shen JJ, Tymkow C, MacMullin N (2005). Disparities in maternal outcomes among four ethnic populations. Ethn Dis.

[105] Shrestha NS, Saha R, Karki C (2010). Near miss maternal morbidity and maternal mortality at Kathmandu medical college teaching hospital. Kathmandu Univ Med J..

[106] Siddiqui SA, Soomro N, Shabih-ul-Hasnain F (2012). Severe obstetric morbidity and its outcome in patients presenting in a tertiary care hospital of Karachi. J Pak Med Assoc.

[107] Tan J, Liu XH, Yu C (2015). Effects of medical co-morbidities on severe maternal morbidities in China: a multicenter clinic register study. Acta Obstet Gynecol Scand.

[108] Tanimia H, Jayaratnam S, Mola GL (2016). Near-misses at the Port Moresby general hospital: a descriptive study. Aust New Zeal J Obstet Gynaecol..

[109] De Mucio B, Abalos E, Cuesta C (2016). Maternal near miss and predictive ability of potentially life-threatening conditions at selected maternity hospitals in Latin America. Reprod Health.

[110] Dias MAB, Domingues RMSM, Schilithz AOC (2014). Incidence of maternal near miss in hospital childbirth and postpartum: data from the birth in brail study. Cad Saude Publica.

[111] Lima HMP, Carvalho FHC, Feitosa FEL (2017). Factors associated with maternal mortality among patients meeting criteria of severe maternal morbidity and near miss. Int J Gynecol Obstet.

[112] Madeiro AP, Rufino AC, Lacerda érica Z, et al. Incidence and determinants of severe maternal morbidity: a transversal study in a referral hospital in Teresina, Piaui, Brazil. BMC Pregnancy Childbirth. 2015;15:1–9. 10.1186/s12884-015-0648-3 PMC4562200 26347370

[113] Galvão LPL, Alvim-Pereira F, de Mendonça CMM (2014). The prevalence of severe maternal morbidity and near miss and associated factors in Sergipe. Northeast Brazil. BMC Pregnancy Childbirth..

[114] Oliveira LC, Ribeiro da Costa AA (2013). Fetal and neonatal deaths among cases of maternal near miss. Rev da Assoc Médica Bras (English Ed).

